# Is intravenous thrombolysis safe for acute ischemic stroke patients taking warfarin with INR 1.9? A case report

**DOI:** 10.1097/MD.0000000000019358

**Published:** 2020-03-06

**Authors:** Zhaokun Li, Jing Su, Shanshan Zhang, Hongcai Du, Yufeng Tang, Jingfeng Duan, Zhonglun Chen

**Affiliations:** a Department of Neurology; b Department of Hematology, Mianyang Central Hospital, Mianyang, China.

**Keywords:** acute ischemic stroke, International normalized ratio, intravenous thrombolysis, Vitamin K

## Abstract

**Introduction:**

Intravenous thrombolysis is not suitable for patients undergoing oral anticoagulants therapy, with INR > 1.7 or PT > 15 s. We described a case of intravenous thrombolysis in a patient with INR 1.9.

**Patient concerns:**

A 66-year-old female patient was diagnosed with acute appendicitis complicated with atrial fibrillation. Seven days after admission, the patient suffered mixed aphasia with right limb asthenia. The NIHSS score was 11 points. and early infarction and hemorrhagic manifestations were not found in the emergency head CT. Thirty minutes after the onset of symptoms, NIHSS of patient increased from 11 to 14, but the INR was 1.92.

**Diagnosis:**

Acute ischemic stroke.

**Interventions:**

The IT therapy was recommended and all the therapy related risks were explained to the patient's parents. Briefly, the patient was given rTPA 38.5 mg. In addition to intravenous thrombolysis, VitK1 40 mg was simultaneously administered.

**Outcome:**

The patient's symptoms of drowsiness were improved. After 24 hours, all symptoms were stabilized with NIHSS of 2 points, there was a slight language obstruction, and no hemorrhagic transformation in head CT. Three months later, the review showed MRS score of 0, and the patient could take care of herself in daily life.

**Conclusion:**

The clinical guidelines are still the main reference for guiding clinical practice, and the main thrombolytic standards and contraindications for treatment still need to be conformed. On this basis, for individualized patients, clinicians must accurately judge the cause of acute stroke, to make optimal choice, reduce disability and mortality, and improve quality of life of patients.

## 1 Introduction

Intravenous thrombolysis (IT) with recombinant tissue plasminogen activator (rtPA) is the only pharmacological therapy that is approved for treatment of the ischemic stroke within 4.5 hours of the stroke onset. Nevertheless, this type of therapy can cause several adverse reactions, such as intracranial hemorrhage, allergic reactions, and potentially fatal intracranial symptomatic bleeding. Therefore, the use of IT requires a rigorous assessment of risk and benefit before it can be approved. According to the *Guidelines for acute ischemic stroke* (AIS) *(2018)* and AHA/ASA guidelines, this therapy is not suitable for patients undergoing oral anticoagulants therapy, with INR > 1.7 or PT > 15 s.^[1]^ If the patients is prescribed VitK1 antagonists (such as warfarin) to maintain an INR > 1.7 or PT > 15 s, the treatment options are very limited. Herein, we reported a single case of a patient with the INR of 1.9 who was taking warfarin and was treated with IT.

## 2 Case presentation

**Figure 1:**
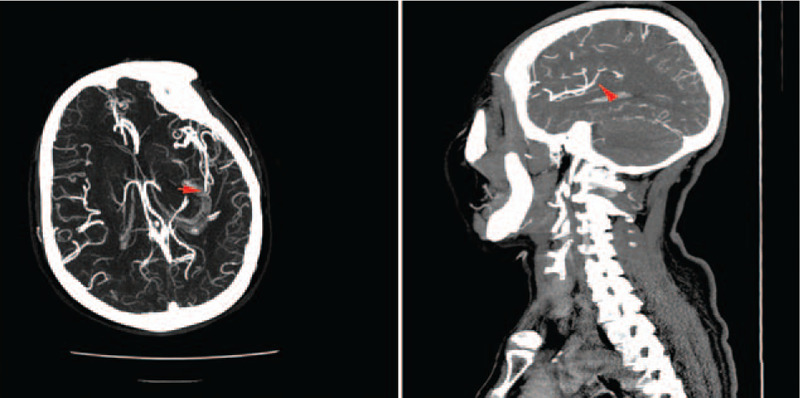
the local lumen in the M3 segment of the left arteriae cerebri media was embolismic.

This study was approved by the Human Ethics Committee of Mianyang central hospital. Informed written consent was obtained from the patient for publication of this case report and accompanying images. A 66-year-old female patient, who was experiencing metastatic right abdominal pain for 2+ days, was admitted to our hospital and was consequently diagnosed with acute appendicitis. The patient had a history of rheumatic heart disease complicated with atrial fibrillation. In addition, she was previously prescribed oral warfarin, had INR of 1.7 to 2.5, and no history of stroke and TIA. The patient was also fully capable of taking care of herself in daily life. After admission, she was prescribed anti-infective and fluid replacement therapy, and continued to orally take 2.5 mg warfarin, while receiving diuresis. In addition, she underwent routine checkup of the heart rate. After admission, her INR was 1.18, and the patient's clinical symptoms were improved and abdominal pain was alleviated. Seven days after admission, the patient suffered mixed aphasia with right limb asthenia, which occurred during dinner time. The NIHSS score was 11 points, and early infarction and hemorrhagic manifestations were not found in the emergency head CT. Thirty minutes after the onset of symptoms, her symptoms continued, and she experienced binocular vision and lethargy, while NIHSS increased from 11 to 14. Next, we evaluated her vascular condition, and the head CTA revealed severe stenosis at the beginning of the M2 segment of the left arteriae cerebri media. Meanwhile, the local lumen in the M3 segment of the left arteriae cerebri media was not developed, which suggested the presence of embolism (Fig. 1). Re-examination showed that platelets were normal, but the INR was 1.92. Consequently, the IT therapy was recommended and all the therapy related risks were explained to the patient's parents. Briefly, the patient was given rTPA 38.5 mg (0.9 mg/Kg) in combination with intravenous thrombolysis at 18:05. Subsequently, the patient's symptoms of drowsiness were improved. In addition to intravenous thrombolysis, VitK1 40 mg was simultaneously administered using another channel. Intravenous thrombolysis was administered 34 minutes from the onset of the symptoms.

**Figure 2:**
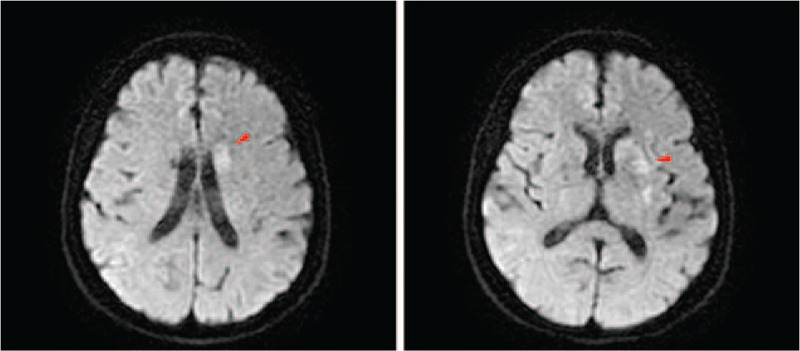
After 24 hours, DWI stimulated acute infarction in the left basal ganglia.

Three hours after therapy, the INR was 1.77. After 6 hours, the patient's clinical symptoms were relieved; she was conscious, her speaking ability was partially recovered, she experienced incomplete aphemia, recovery of right limb muscle strength and NIHSS of 6 points. After 24 hours, all symptoms were stabilized with NIHSS of 2 points, there was a slight language obstruction, and no hemorrhagic transformation in head CT. DWI stimulated acute infarction in the left basal ganglia (Fig. 2). At discharge, NIHSS was 1 point, while the MRS was 1 point. Three months later, the review showed MRS score of 0, and the patient could take care of herself in daily life. She continued oral warfarin treatment.

## 3 Discussion

In China, the incidence of ischemic stroke is increasing year by year, and the fatality/ disability rate is 34.5% to 37.1%,^[2]^ which exerts huge medical economic burden and spiritual burden to society and families. Nevertheless, among patients with AIS who are eligible for IT, less than 3% are suitable candidates for the IT therapy. Due to various contraindications such as thrombocytopenia and INR prolongation occurring in the very limited time window in which the therapy can be applied, patients are unable to receive intravenous thrombolysis, thus missing a valuable treatment, and potentially facing the disability. Nevertheless, previous studies have suggested that the reduction of thrombolysis restrictions can increase the thrombolytic proportion in emergency patients.^[3]^ Hence, expanding the indications for intravenous thrombolysis is important for increasing the proportion of IT.

It is uncertain whether thrombolytic therapy is safe in patients with AIS treated with warfarin. IT may increase the risk of symptomatic intracranial hemorrhage and can even lead to death; therefore, it is not recommended for patients with INR >1.7.^[1,2]^ Thus, the risks such as severe consequences due to stroke or intracranial hemorrhage due to thrombolysis, as well as benefits should be adequately considered in the evaluation of patients with thrombolysis and elevated INR. Nevertheless, this does not mean that IT therapy cannot be implemented in patients with extended INR. Mazya et al have found that IT is safe and effective in patients receiving oral warfarin. They also found that a recanalization rate was significantly higher in patients with INR < 1.7 compared to the control group.^[4]^ Yet, it remains unclear whether the safety of intravenous thrombolysis in patients with INR > 1.7 can be reduced. Italo et al have reported on 2 acute cardiogenic stroke patients receiving warfarin who had INR > 1.7 and 1.9, respectively, and were consequently treated with intra-arterial thrombolysis. Both patients recovered well and did not develop any intracranial hemorrhage.^[5]^ Furthermore, a study based on SITI registration in Europe demonstrated that among 212 patients who received oral anticoagulant drugs in combination with IT, 45 patients had an INR > 1.7. After adjusting the independent predictors, these patients did not show significant differences in terms of symptomatic intracranial hemorrhage rate, 3-month mortality and prognosis compared with patients who did not take anticoagulant drugs.^[6]^ Moreover, Frank et al^[7]^ have found that among 2755 thrombolysis patients enrolled in large clinical studies based on VISTA data, 138 cases had an INR >1.7, and the increase in INR in 14 patients was due to oral warfarin. The results showed that patients receiving oral warfarin did not develop intracranial symptomatic hemorrhage, and only 7 of 138 patients developed intracranial symptomatic hemorrhage. After adjusting for age and NIHSS baseline score, the patients with an INR > 1.7 showed a slightly higher probability of good prognosis than the control group; nevertheless, the difference was not statistically significant. In a clinical study conducted in the United Kingdom, only 1 of the 115 stroke patients with INR > 1.7 receiving warfarin developed intracranial symptomatic bleeding,^[8]^ which suggests that IT may be effective and relatively safe in stroke patients with an INR >1.7.

In clinical practice, for AIS patients with an INR > 1.7, IT should reduce the risk of hemorrhage and improve the prognosis rate. Using cardiogenic stroke animal models, Sun et al^[9]^ have attempted to reverse the increase in INR after taking warfarin using PCC, followed by IT therapy with rTPA. The results showed that PPC reduced the incidence of severe bleeding after IT in rats with an INR of 2.0 to 3.0 caused by warfarin, which suggested that IT therapy might be given after reversing INR with PPC in patients receiving anticoagulation therapy. Based on this important finding, Jalini et al^[10]^ have reported a case that was treated with IT following PCC to quickly reverse INR. Symptomatic intracranial hemorrhage did not occur after thrombolysis, and the NIHSS score was reduced from 24 points at admission to 6 points at discharge; and was further reduced to 4 points after 8 weeks. However, since PCC contains coagulation factors II, VII, IX, X, and proteins C and S, it may increase the risk of thrombosis by 1% to 2% in patients with stroke. Hence, PCC is not recommended for patients with AIS.^[11,12]^ Meanwhile, Vitamin K1 can activate the existing clotting factors in the body without the need for synthesis or supplementation of new proteins, and can reverse the abnormally elevated INR caused by warfarin. It does not promote thrombosis under conventional treatment, and intravenous injection can accelerate the effect of reversing INR.^[10–12]^ It is also used to reverse the increase in INR caused by warfarin during the perioperative period of surgery, so as to reduce the risk of bleeding in emergency surgery.^[13]^ Invasive emergency surgery should be performed at 6 to 12 hours after intravenous administration of Vitk1 10 mg. However, for patients receiving warfarin with an INR at the treatment window (2.0–3.0) and below, the proportion of spontaneous bleeding is very low. Thus, in the case of non-invasive conditions, VitK1 before thrombolysis may have a special effect on patients taking warfarin who have an INR below the standard.^[12]^

In this case study, all the clinical examinations and preliminary imaging evaluations were completed within 1 hour of the onset, and no other intravenous thrombolysis contraindications were found except INR, which led us to rethink the thrombolytic indications. Initially, the IT therapy was rejected because it was believed that the risk of bleeding was greater than its clinical benefit would be due to an INR of 1.9. However, during the re-evaluation, it was found that the patient was receiving warfarin, and that she regularly underwent INR test, which was maintained between 1.7 and 2.5. Before the visit, the patient did show symptoms of ischemic stroke or TIA. In terms of the risk of stroke, in addition to the common causes of arteriosclerosis, atrial fibrillation, and heart valve disease, there are other rarely found causes, such as inflammation, tumors, PFO, vascular malformations, and immunity. The patient in the present study was admitted due to acute appendicitis. It was unclear whether the stroke occurred due to the inflammatory response that activates another coagulation pathway, or due to an abnormal embolism of a small bacterial embolus. The CTA showed severe stenosis in the left arteriae cerebri media, with partial occlusion at the distal M3 segment, which indirectly suggests that the stroke may not be caused by a cardiac arrest. With the continuation of the patient's clinical symptoms, the thrombotic mechanism was re-analyzed, and risks and benefits were re-assessed, which prompted deep and full communication with the family again. Subsequently, the patient was given IT therapy. In the meantime, in order to rapidly neutralize the elevation of INR caused by warfarin, 40 mg VitK1 was synchronously infused.

Recently, Kefas et al^[14]^ have reported a case with INR 1.9 due to warfarin, who was treated with IT. Nonetheless, the patient was confirmed with cardiogenic stroke, and was not given drugs (such as PCC, warfarin and FFP etc.) to reverse INR before and during intravenous thrombolysis. According to our knowledge, this was the first case report in which intravenous thrombolysis was given at an INR of 1.9 due to warfarin, and Vit k1 was simultaneously given to reverse the warfarin-induced INR elevation. Although it is a case study, it may also be a breakthrough window. Previous studies were not specifically designed for stroke patients with INR > 1.7. Purely extracting part of the population from these studies will inevitably cause some defects in terms of sample size, assessment method before thrombolysis and intervention strategies. Therefore, it is necessary to perform large-sample clinical studies on implementing thrombolysis in patients with INR > 1.7, to re-evaluate the effectiveness and safety of thrombolysis, as well as to evaluate the effect of drugs for reversal of INR on thrombolysis.

In clinical practice, not every stroke patient with an INR > 1.7 can be treated with IT. Currently, the clinical guidelines are still the main reference for guiding clinical practice, and the main thrombolytic standards and contraindications for treatment still need to be conformed. On this basis, for individualized patients, clinicians must accurately judge the cause of acute stroke, to make optimal choice, reduce disability and mortality, and improve quality of life of patients.

## Author contributions

**Conceptualization:** Zhonglun Chen.

**Data curation:** Zhaokun Li.

**Formal analysis:** Zhaokun Li, Jing Su, Jingfeng Duan.

**Investigation:** Zhaokun Li, Jing Su, Shanshan Zhang, Yufeng Tang.

**Methodology:** Zhaokun Li, Jing Su, Yufeng Tang.

**Project administration:** Yufeng Tang, Zhonglun Chen.

**Resources:** Shanshan Zhang, Zhonglun Chen.

**Software:** Jing Su, Shanshan Zhang, Hongcai Du, Jingfeng Duan.

**Supervision:** Shanshan Zhang, Hongcai Du, Jingfeng Duan.

**Validation:** Hongcai Du.

**Visualization:** Hongcai Du.

**Writing – original draft:** Zhaokun Li.

**Writing – review & editing:** Jing Su, Shanshan Zhang, Hongcai Du, Yufeng Tang, Jingfeng Duan, Zhonglun Chen.
